# Finding Hope in Finnish Parents Following the Traumatic Death of Their Child

**DOI:** 10.1177/00302228241272553

**Published:** 2024-09-06

**Authors:** Nur Atikah Mohamed Hussin, Anna Liisa Aho, Jari Kylmä

**Affiliations:** 1 Faculty of Social Sciences (Health), 7840 Tampere University, Tampere, Finland

**Keywords:** parents, grief, traumatic death, hope, coping

## Abstract

Hope is a complex and ever-evolving personal phenomenon that plays a vital role in individuals’ abilities to cope with stressful events. This is particularly true for parents who are coping with the traumatic loss of a child. However, the topic of hope in this context is often inadequately addressed. The primary objective of this paper is to gain insight into the hope held by Finnish parents following the traumatic loss of a child. This qualitative study unfolded in two phases. A total of 117 participants took part in the study, including 108 females, 5 males, and 4 individuals who chose not to disclose their sex. Subsequently, 17 parents participated in in-depth phone interviews. Thematic analysis was conducted to identify key themes. Several themes emerged from the analysis, including the endurance of hope amidst uncertainty, the hope for a reunion based on faith, hope directed towards family members, and moments of hopelessness regarding the future. The findings of this research are pivotal in enhancing our comprehension of the challenges faced by grieving parents in the aftermath of a child’s traumatic death. Moreover, this study holds significant relevance for professionals who work with bereaved parents following the traumatic loss of a child.

## Introduction

The parent-child relationship is widely regarded as the most significant bond that spans a person’s lifetime (Frosch et al., 2019). This unique connection is characterized by the deep emotional ties between parents and their children (Arnold & Gemma, 2008), which begins to form even before the child is born, during the mother’s pregnancy (Prior & Glaser, 2006). Parents view their children as an extension of themselves, embodying their aspirations, needs, and desires for a lasting legacy (Rando, 1986). Conversely, children symbolize the continuation of their parents’ lineage and represent hope for the future. Particularly, they are expected to carry on the family name, outlive their parents, and fulfill their parents’ dreams.

The death of a child is a profound loss that represents the end of future possibilities, hopes, and dreams (Arnold & Gemma, 1994). A study on mothers who experienced perinatal deaths found that they faced challenges in holding onto their hopes for a new life (Bailey et al., 2019; Nuzum & Meaney, 2018). Nevertheless, these mothers recognized the significance of hope and made efforts to sustain it even after their loss (de Andrade Alvarenga et al., 2021). Hope serves as a valuable resource for individuals during times of suffering (Holtslander & Duggleby, 2009). Within the existing literature, hope is reported as an important resource during times of suffering (Holtslander & Duggleby, 2009) and a key factor for recovery when facing trauma, such as the death of a loved one, as in suicide (Huen et al., 2015). Hope can serve as a resilience factor in mitigating the impact of hopelessness, which is associated with a higher risk of suicide (Huen, 2015), depression (Liu et al., 2015), and complicated grief (Toedter et al., 2001). A high level of hope is predictive of positive adaptation to life after bereavement (Chow, 2010; Cutcliffe, 1998; Einav & Margalit, 2020). Nonetheless, the literature on hope and grief is still limited, considering that most literature on traumatic grief focuses solely on grief following suicide (Huen, 2015).

To date, there is a lack of understanding regarding hope and parents following the traumatic death of a child. Most studies have focused solely on general types of loss in general populations (Chow, 2010; Cutcliffe, 1998; Einav & Margalit, 2020). Furthermore, previous studies have primarily utilized quantitative methods (e.g., Bellini et al., 2018; Chow, 2010; Einav & Margalit, 2020), which may limit the depth of data on bereavement and hope. Additionally, these studies have primarily focused on hope-based interventions for bereaved individuals (e.g., Cutcliffe, 1998; Cutcliffe, 2004), and hope in individuals with chronic illness (e.g., Baczewska et al., 2020; Kylmä et al., 2001; Sabri et al., 2023).

This study aims to explore the concept of hope in parents who have experienced the traumatic death of a child. By doing so, it seeks to enhance our understanding of the complexity of bereavement in parents and emphasize the significance of cultivating hope in grief intervention. The prospect of personal growth following a devastating loss not only provides hope to the bereaved individual but also equips them with the knowledge of how their life has been altered and the means to begin reconstructing it.

### Traumatic Death

Following the traumatic death of a child, parents are at risk of experiencing a range of negative psychological outcomes, including complicated grief (Kersting et al., 2011), anxiety, depression, post-traumatic stress disorder (PTSD) (Morris et al., 2019), potentially leading to increased mortality rates (Davies, 2006). Subsequently, parents can be at risk of experiencing prolonged grief that can be intense persistent grief that causes problems and interferes with daily life (Hussin et al., 2018). Traumatic deaths, including homicide, suicide, accidents, natural disasters, terrorism, or warfare (Rynearson, 2006), exacerbate these difficulties due to their distressing nature, suddenness, and sometimes intentional harm or grotesqueness. Furthermore, considering the corresponding characteristics and definitions of traumatic deaths, we extended our study to encompass parents who have endured traumatic losses resulting from medical errors and drug-induced deaths.

To add to more challenges, parents who experienced the traumatic death of their child also have to navigate legal proceedings, involvement with the criminal justice system, and media attention (Herber, 2016). These factors contribute to the severity of psychological distress, including post-traumatic stress disorder (PTSD), depression, and anxiety (Sprang, 1997). Despite the profound impact of traumatic loss, parents must navigate their grief effectively to foster positive outcomes. Various factors contribute to coping among grieving parents, such as social support, optimism, and perceived control (Prati & Pietrantoni, 2009). Moreover, studies often highlight hope and/or optimism as significant elements in coping strategies and problem-solving skills (Smithson et al., 2022). However, while hope and/or optimism have been sporadically discussed in conjunction, the role of hope specifically in facilitating coping among bereaved parents remains relatively understudied.

### Finnish Background

Over the past few decades, Finland has experienced a marked shift in the religious influence of the Evangelical Lutheran Church (Butters, 2023). In the 1980s, religion played a significant role in the everyday lives of Finns. However, its influence has waned considerably in recent years. Concurrently, as Finnish society has become more multicultural, attitudes toward death have evolved and become more nuanced. While traditional rituals and funerals are still observed, Finns have developed more sophisticated ways of coping with grief and preserving memories and hope after the loss of loved ones.

Research on hope in the context of Finnish parental grief is relatively sparse. A notable study by Keskinen et al. (2019) found that bereaved parents often used Facebook to share photographs and write about the positive aspects and joys of daily family life on discussion boards. Additionally, these parents expressed through photographs their hope of reuniting with their deceased children in the future (Irwin, 2015). This study highlights that the grieving culture among Finns is a complex and meaningful subject, deserving of further scientific exploration.

### Aims

This current study is aimed at understanding parents’ hope following the traumatic death of their child.

## Methods

### Research Design

This study utilized a descriptive qualitative design to understand parents’ hope following the traumatic death of their child. A descriptive qualitative design was utilized to describe details of a phenomenon from those with experience of the phenomenon (Bradshaw et al., 2017). The concept of hope provides a beneficial framework for understanding parents’ hopes following the traumatic death of their child.

### Data Collection and Participants

Participants for this study were identified through purposive sampling, focusing on parents who lost a biological child to a traumatic death. The age of the deceased children’ age was not limited in the study. The demographic profiles of the participants are presented in Table 1.

**Table 1.** List of Demographic Characteristics of the Participants From the LimeSurvey Questionnaire.

Background variable	*n* (117)	%
Sex (*n* = 117)		
Female	108	92.3
Male	5	4.3
Do not want to disclose	4	3.4
Age in years (*n* = 117)		
<40	11	9.4
40–49	29	24.8
50–59	53	45.3
≥60	21	17.9
Do not want to disclose	3	2.6
Marital status (*n* = 117)		
Married	73	62.4
In a relationship	19	16.2
Not in a relationship	25	21.4
Place of residence in Finland (*n* = 117)		
Southern	45	38.5
Western	36	30.8
Eastern	18	15.4
Northern	18	15.4
Educational background (*n* = 117)		
Elementary/High school	51	45.6
Upper secondary vocational qualification	10	8.5
University of applied sciences degree	25	21.4
Academic degree	31	26.5
Employment status (*n* = 117)		
Employed	81	69.2
Unemployed	17	14.5
Other	19	16.2
Member of a religious community (*n* = 117)		
Yes	48	41.0
No	69	59.0
Perceived health status (*n* = 117)		
Good	34	29.0
Satisfactory	43	36.8
Bad	35	29.9
Do not want to be disclosed	5	4.3
Number of surviving children (*n* = 117)		
0	10	5.5
1 2	71	60.7
≥3	36	30.8
Cause of death of a child (*n* = 117)		
Drowned	7	6.0
Homicide	7	6.0
Medical errors	3	2.6
Other types of accidents such as accidents while horse riding, in construction areas, in baby cots, etc.	17	14.5
Drug-induced deaths	3	2.6
Suicide	38	32.5
Unknown	5	4.3
Vehicle accident	40	34.2
Time elapsed since the death of the child (in years) (*n* = 117)		
<1	20	17.1
1 5	63	53.8
≥6	34	29.1

An online advertisement for the study was posted on the websites of Finnish bereavement organizations, their member mailing lists, and closed discussion groups. The advertisement provided information about the study’s objective and purpose, contact details, and a link to access the electronic questionnaire using LimeSurvey. Additionally, a cover letter was distributed to participants along with the survey. Participants who met the inclusion criteria were invited to take part in the study (Table 2).

**Table 2.** List of Demographic Characteristics of the Participants From the Interview.

Theme	Categories	Number (*N* = 17)
Sex	Female	15
	Male	2
Cause of death of a child	Suicide	7
	Accident	5
	Homicide	1
	Medical errors	2
	Sudden deaths	2

The LimeSurvey platform was used to develop an electronic questionnaire, which is available in both Finnish and English. The questionnaire includes questions about demographic background variables such as age, sex, cause of death, duration since loss, number of surviving children, perceived health status, and details of consent. In addition, there are open-ended questions that explore parents’ feelings of hope after the traumatic death of their child. After completing the electronic questionnaire, participants were given the option to express their interest in a telephone interview. Those who indicated interest and agreed to participate in the interview provided their contact information. Subsequently, researchers contacted the participants via email or text messages to arrange a convenient interview time.

The telephone interviews were conducted in Finnish and consisted of the same set of open-ended questions as featured in the electronic questionnaire. The purpose of these interviews was to provide participants with an opportunity to elaborate on their responses from the electronic questionnaire, which they had completed prior to the interview. After obtaining consent from the participants, the interviews were recorded. Upon concluding the interview, the interviewer switched off the recording device and engaged in an informal conversation with the parents. The duration of the interviews ranged from 45 minutes to 1.5 hours.

As a result, 117 participants submitted their answers via electronic questionnaires, and 17 participated in telephone interviews. The data collection period spanned from November 2022 to March 2023.

### Trustworthiness

Aspects of research trustworthiness are typically described using four key factors: credibility, confirmability, dependability, and transferability (Guba & Lincoln, 1989). To ensure the credibility of the study, debriefing and member checking were conducted. All researchers were involved in the member-checking process. A purposive sampling method was employed to deliberately select participants who could provide rich information about their experiences of losing a child due to traumatic death, thus ensuring the transferability of this study to similar populations. To ensure the confirmability of this study, findings were transcribed verbatim without the influence of the researcher’s biases, motivations, or perspectives. Dependability was achieved through a detailed description of the methodology used in this study. Triangulation of the study was achieved through incorporating individual interviews, field notes, and voice-recorded data. The researcher compared the notes with the recordings multiple times and compared the emergent findings to existing literature and theory in the field.

### Researchers’ Reflexivity

Reflexivity is crucial for researchers as it involves recognizing and clarifying how their personal experiences and positionality influence their analytical framework, ensuring they remain aware of their involvement in the research process (Mason, 2018). The data collection was conducted in two phases. The first phase involved an electronic questionnaire. This method minimized the researcher’s influence on participants, as it was conducted passively without any active participation in discussions. Utilizing an online platform for data collection reduces the risk of human error and eliminates the potential influence of the researcher, thereby reducing sources of bias (Emery, 2014). However, the researcher’s interpretative influence remains comparable to other qualitative research approaches when presenting these findings.

The second phase involved telephone interviews conducted by a single researcher. She is a Finnish woman fluent in English, with personal experience of miscarriage. Throughout the interview process, she kept a reflective journal to bracket her personal values, emotions, thoughts, and potential biases, as suggested by Rolls and Relf (2006). Bracketing helps mitigate any preconceptions arising from direct experience that may affect the research process. Additionally, peer debriefing was utilized to validate interpretations and reduce researcher bias, as recommended by Lincoln and Guba (1985).

The first author, an Asian female postdoctoral researcher, has been conducting grief-related research for eight years. The third author, a male Associate Professor, is an expert in hope-related topics across various populations. Both served as peer debriefers. The first and third authors’ lack of previous experience with grief helped them monitor the second author’s subjectivity, ensuring the elimination of biases or misinterpretations during data collection and analysis.

### Ethical Approval

This study focused on sensitive topics in social research, specifically involving bereaved parents. Therefore, obtaining ethical approval was crucial to safeguard the dignity, rights and welfare of the research participants (World Health Organization, 2023). The Tampere University ethics committee approved this study (76/2022).

Before participating in the study, all participants received comprehensive information about its purpose and were required to provide both online and verbal consent. Only after completing the consent process did, they proceed to answer the online questionnaire and participate in interviews. It is worth noting that all participants granted their consent before becoming part of the study.

To ensure confidentiality, the study was conducted anonymously. Demographic data is securely stored on the encrypted LimeSurvey platform. Additionally, all collected data will be promptly and securely destroyed upon the conclusion of this study.

After the interviews, the interviewer inquired about the participants’ well-being and their reflections on the interview experience. Furthermore, the interviewer offered supportive guidance to the parents, should they require it, and encouraged them to promptly reach out if they felt the need for assistance.

## Data Analysis

The open-text responses from the electronic LimeSurvey survey were initially transferred to an Excel database for analysis. Exporting responses into a compatible database helps eliminate transcription errors and prevents participants from modifying their responses (Ilieva et al., 2002). A professional translator then translated the data from Finnish to English. Similarly, the interviews were transcribed and translated from Finnish to English by a professional translator.

The data from both sources were combined into a single document and then analyzed. A thematic analysis was conducted, following the six-step process outlined by Braun and Clarke (2006). This process includes stages of familiarization, coding, generating themes from the codes, reviewing themes, defining and naming themes, and writing up. After becoming familiar with the data, the transcripts were analyzed line-by-line, and codes were generated inductively. Codes with similar meanings were then clustered to develop preliminary themes, which were iteratively reviewed and refined.

## Results

The themes that emerged from the data analysis are finding hope in the face of uncertainty, the hope of faith to be reunited, hope dedicated to family members, and being hopeless about the future.

### Finding Hope in the Face of Uncertainty

After experiencing the devastating loss of a child, parents often strive to hold onto hope in their present lives. The ability to cope with this profound loss enables parents to embrace a more optimistic outlook for their future. In fact, parents shared that despite their child’s passing, they continue to find inspiration in the perspectives on life and death that their deceased child had. This reflection serves as a source of hope for parents as they navigate the aftermath of their loss. A 53-year-old father who lost his child due to an accident shared his thoughts on hopes after his loss. He shared:

Right now, I feel hope. Perhaps stronger than ever before. Two big factors affect the situation. 1. recovery from previous burdens, and 2. the effects of (child's name) life and death on the way we see life and thus hope. I would see that there have been some changes in what is hoped for, how she saw it, and how that hope can be achieved. So, you could say that at first hope was lost, but now it has returned even stronger. At the same time, its content has changed. Likewise, the fact that I can influence whether I achieve my hope and how I achieve it.

For parents, sunshine represents hope. The transition from a dark season to a season with more sunshine helps parents understand that having hope is possible even after the death of a child. A 56-year-old mother who lost her child due to suicide described:

There was an anniversary in autumn and then I felt that I no longer had any hope, that my sun would not rise again. Now that the days are longer, the light has brought hope back. I can see into the future again.

The parents faced the daunting challenge of holding onto hope. The prospect of being reunited with their departed loved one and the lingering sense of their presence provided solace and strength, enabling the parents to persevere in their daily lives. Remarkably, even those close to the grieving parents attested to experiencing signs and dreams involving the deceased child, further affirming a palpable connection between the living and the departed. A 65-year-old mother who lost her child due to an accident during a horse ride maintained:

Finding hope has been difficult. The hope of a reunion and the feeling of our son's presence are the most important feelings of hope. I also hope to see “signs” or dreams of my son that would concretize hope and presence. At first, I felt I had received these signs, and others close to me also talked about them.

### Hope of Faith to be Reunited

For some parents, their religion provides them with the faith and hope that they will be reunited with their deceased child in the afterlife. A 56-year-old mother who lost her child due to homicide said:

The hope of seeing you again heightened. Only by believing in Jesus can one live even if one is dead. It's such a significant thing, and it's become really important to know.

Similar to a 58-year-old mother who lost her child due to an accident, she believed that her child’s soul lives on. She also shared her hopes to meet with the deceased child again. She remarked:

His soul is out there somewhere. It is good to live in the hope of seeing [him] again. Love is great!

For some parents, the death of their child has led to a deepening of their spirituality. They may not feel the need to dwell on the specific causes of death or seek a complete understanding of the situation. Instead, they find solace in reading and listening to materials that can assist them in coping with their loss. These parents prioritize focusing on their present lives and nurturing new hopes, such as the possibility of being reunited with their deceased child. Their faith in their religion provides them with hope and the belief that they are not alone in their journey. A 39-year-old mother who lost her child due to an accident mentioned:

I am more sensitive and spirited after the loss. It seems necessary to try to find explanations for what happened. I've been thinking more about the time after death, and I've started to hope and maybe trust in a reunion, and that my child is still present in our lives in some form. I have also searched for more experiences of others to read or listen to. It feels more certain when you know that you are not alone with hope and faith.

The reunion with the deceased child is also described as being together as a family again. A 65-year-old mother shared:

Hope only for a reunion! Of course, we also hope for the time spent with the family...!

### Hope is Dedicated to Family Members

For some parents, their focus shifts to the remaining children in the family. They see their role as parents as crucial in maintaining the family’s functioning after the tragic loss of a child. A 37-year-old mother who lost her child due to an accident remarked:

There is always hope. The future is in the little sisters. In the middle of the depressive symptoms, there was a feeling of hopelessness, but as the feeling improved, hope also returned.

Parents also saw themselves as responsible for carrying on the hopes of the deceased child to other family members. They recognized that the death of the child had impacted the entire family, and emphasized the importance of ensuring that the remaining children could lead happy lives despite the absence of their siblings. A 50-year-old mother who lost her child due to an accident said:

I just hope I go after my daughter. That her life must be according to the deceased brother who wanted her to be happy. This is the only way to make sure that the deceased brother is happy. Her brother's death has affected her so much that I wish for happy moments in her life.

Likewise, some parents reported that over time, their grief gradually decreased and they became more optimistic about the future. They hope that their remaining children will be able to pursue their aspirations and become the individuals they want to be. While the children acknowledge the difficulty of losing their siblings, they also maintain a positive outlook on their own future. The parents also strive to establish trust with their children, serving as a source of support. A 48-year-old mother who lost her child due to suicide maintained:

For a long time, there were only small glimmers of hope. Now, gradually, 3.5 years after the death of my child, I am cautiously optimistic about the future. I don't expect much from myself, as long as I can live a safe life like I do now. There is a lot of hope for my living children. Both are studying their chosen field, and despite the loss they have experienced, have a forward-looking attitude. I hope that we have a good long life and that the children do well.

In addition, some parents find hope in their grandchildren. These grandchildren serve as a source of motivation for the parents, inspiring them to believe in their present and future. Consequently, the parents become more inclined to prepare themselves for potential future scenarios and adjust to any losses they may experience. A 65-year-old mother who lost her child due to suicide shared:

I see hope and a future in my children, and especially in my grandchildren. They always make me believe in life and the future. My future is full of possibilities, as long as I slowly move forward in my life.

### Being Hopeless About the Future

Despite the possibility of finding hope after the traumatic death of their child, some parents reported feeling a diminished sense of hope for their future. The sudden and traumatic loss of their child has left these parents uncertain about their own lives and has made them feel unsafe. A 50-year-old mother who lost her child due to an accident said:

In addition, (when basic security has been so drastically shaken) one does not dare to hope for anything more when you know that your loved one can be taken away in the blink of an eye.

Likewise, some parents, especially those who have lost their children to suicide, associate this loss with a loss of hope. These parents believe that the death of their child is connected to their own loss of hope, and they feel that they have no right to hold onto hope. A 63-year-old mother who lost her child due to suicide noted:

There is no hope! I have no hope! A child who commits suicide has no hope, so his mother can't have it either. Hope is fucked.

Furthermore, some parents expressed that the death of their child has transformed them from hopeful to hopeless. They have also become individuals who no longer care about their own life and death. A 43-year-old mother who lost her child due to an accident remarked:

My life feels pointless, I used to be more hopeful. I never thought about the possibility of burying my child, this has changed me a lot, I don't care about life, but I don't want to die either.

## Discussion

This qualitative study aims to provide insight into the experience of hope in parents who have lost a child in a traumatic event. The findings suggest that despite the immense pain and sorrow associated with the death of a child, parents can still find hope during difficult times. As part of the grief recovery process, parents must make efforts to preserve their hope and a positive outlook for the future (Bailey et al., 2019). Therefore, in this current study, parents are required to face their grief and trauma while continuously preserving hope throughout their grieving journey.

This study discovered that parents were able to find hope through religious coping. For parents who are dealing with the loss of a child, religious coping serves as a mechanism to reignite the hope of reuniting with their departed loved ones and helps them cope with their grief (Hussin et al., 2018). Although there is limited research on reuniting with the deceased and post-grief growth (Čepulienė & Skruibis, 2022), the parents in our study consistently reported that their beliefs enable them to cope with their losses and maintain a positive outlook on the future through spiritual means. Positive religious coping is closely linked to a heightened level of hope (Shamsalinia et al., 2015). By focusing on God and nurturing hope, parents demonstrate greater resilience in the face of life’s challenges (Tarakeshwar et al., 2006).

This study also highlighted parents who found hope through continuing bonds with their deceased child. Some parents believe that their deceased child exists in a spiritual form, providing them solace and comfort. The occurrence of “hallucinatory experiences” is particularly common, especially after an unexpected death or the loss of a young person (Baumeister et al., 2017). Parents explicitly believe that the relationship between them and the deceased persists in spiritual ways, helping them find hope for their future, including the possibility of being reunited and becoming a family again.

Additionally, this study revealed that parents are aware that the loss not only affects them but also has an impact on all other family members. Grief is a family affair (Breen et al., 2019). Following the traumatic death of a child, family members collectively work as a system to cope and adapt to the losses. Consequently, parents not only strive to find their own hope but also play a crucial role in fostering hope in other family members. Parents view the endeavor to sustain hope within the family as a collective effort. Rather than solely concentrating on their individual hopes and futures, the parents in this study regard their post-traumatic loss life goals as being centered on the unity and well-being of the entire family. Consequently, the parents in this study report offering support to their family members to assist them in sustaining hope and fostering optimism for their shared future as a family. Through providing support to their family members, parents collaboratively strive to achieve the shared objectives of finding hope and cultivating optimism for their future as a family unit.

Most literature focuses on post-growth and hope (e.g. Chow, 2010; Cutcliffe, 1998; Einav & Margalit, 2020). However, it is important to acknowledge parents who may struggle to find hope after the traumatic death of their child. This current study reported that parents may have feelings of guilt and self-blame due to the traumatic death of their child which can hinder them from maintaining their hope and finding hope after the loss. The feelings of guilt and blame are common responses among parents after the traumatic death of their child (Frei-Landau et al., 2023). Although, literature reported that individuals with a sense of hopelessness are at risk of contemplating suicide (Snyder, 2002), this current study does not support the findings. Nonetheless, this study has no intention to study this relationship in detail. Nevertheless, this current study echoes the potential for parents to feel hopeless following the traumatic death of their child. Therefore, considering referring these parents for appropriate intervention is essential. According to a recent meta-analysis of Norwegian literature on recovery-oriented practices by Klevan et al. (2021), fostering hope involves helping clients build self-belief, trust in others, and the ability to recognize and embrace future opportunities. This study highlights the potential of implementing suitable interventions to support parents in rekindling hope after the traumatic loss of a child.

### Limitations of the Study

The study’s recruitment involved posting advertisements on peer-support websites in Finland. As a result, parents who did not have access to these websites might not have been able to take part in the study. Nevertheless, using an online platform for advertising allowed a more extensive and geographically diverse group of interested parents to participate. Additionally, the study employed LimeSurvey as its data collection tool, which could have posed challenges for parents lacking Internet access. To mitigate this limitation, the research team conducted phone interviews with parents who expressed interest, aiming to minimize potential exclusion and create a more inclusive participation process. This study also employed electronic surveys and phone calls for data collection, which limited the researcher’s ability to document field notes from informal conversations. While this represents a drawback of using these methods, electronic surveys and phone calls are essential due to their flexibility, ability to ensure anonymity, and elimination of geographical barriers. Additionally, given that bereaved parents are a sensitive population, electronic surveys allow parents to respond freely without fear of recognition or judgment. Furthermore, telephone interviews can create a more balanced power dynamic between the participant and the interviewer (Vogl, 2013). The perceived distance provided by phone calls also makes it easier for participants to take control and end a conversation that may not feel comfortable. Furthermore, this study does not account for factors such as the child’s age, which could influence the parents’ experiences. The child’s age is not included in the data analysis for this study. Consequently, further research is required to explore these factors in greater depth.

## Conclusion

Our findings underscore the significance of hope in parents after the traumatic death of their child. Despite the devastating loss of a child, parents try to find hope and maintain optimism about their future. Several factors such as being reunited in the future and other family members are important in helping parents find hope. Furthermore, parents play a crucial role in fostering hope and optimism among other family members. Especially, rather than seeing the maintenance of hope as an individual work, parents collectively put effort into fostering hope and optimism toward their future together.

However, it is important to acknowledge that for some parents, finding hope and optimism in the aftermath of such a tragic loss can be an arduous challenge. Some parents may find themselves struggling with feelings of hopelessness, which can lead to adverse outcomes, including the risk of suicide. Therefore, it is imperative to offer support to parents in the aftermath of a traumatic loss, considering the circumstances surrounding the death.

Though hope is important for better grief outcomes in parents, it is equally important to acknowledge parents who may not be able to find hope after the traumatic death of a child. According to Wand et al. (2022), collaborative work, treating individuals who feel hopeless equally, optimism and optimism toward recovery can inspire hope in individuals who feel hopeless. Therefore, this study suggested that social workers should recognize hope as an important factor in parents’ grief journey. This way, social workers can empower individuals to grieve healthily.
